# Supplementary material for: Extracellular matrices of stromal cell subtypes regulate phenotype and contribute to the stromal microenvironment in vivo
Source: Stem Cell Res Ther. 2024 Jun 18;15:178. doi: 10.1186/s13287-024-03786-1 (PMC11184721; doi:10.1186/s13287-024-03786-1)
Supplement: Supplementary file 1 — Supplementary Figure 1: A Quantification of senescence associated beta-galactosidase staining at 72 hours. B Heatmap of microarray-derived data showing Log2 fold-change in expression of genes identified in the Reactome_senescence genes database in Y201 and Y202 compared to their parental cells. The classical senescence-associated genes CDKN1A, CCL2 and TP53 were not detected by the microarray and are not represented in the heatmap. C Outline of the CellProfiler pipeline for detection and quantification of crystal violet stained CFU-F assays. Supplementary Figure 2. A Quantification of focal adhesion size following fluorescent staining of Y202 cells treated with either Y201-CM or Y202-CM for 24 hours. B Mean colony area from in vitro aged primary BMSC CFU-Fs (n=5) (ANOVA: F = 3.863, df = 1.833, 7.332, p = 0.0738) C Total colonies identified from CFU-Fs of in vitro aged primary BMSCs (Friedman test, p = 0.0085) with post-hoc test revealing significant effect of Y201CM vs no CM (p = 0.0044) n = 5. *p≤0.05, ***p<0.001, ns = not significant. Error bars ± SEM. Supplementary Figure 3. A Significantly enriched proteins secreted by Y202 versus Y201 represented in order of normalised abundance from LC-MS/MS. Graphs are split for ease of interpretation while maintaining a linear scale, Means ± SEM. B Matrisome annotated proteins that were not significantly altered between Y201 and Y202 secretomes. Proteins are labelled as core-matrisome (blue) or matrisome-associated (red) with shading representing sub-categories of these annotations. Supplementary figure 4. Representative immunofluorescence microscopy images of from n=4 mice various ECM proteins (violet) in mouse bone marrow with DAPI (blue) nuclear stain. Top row shows imaging of epiphyseal region of mouse femur. Middle row shows regions from the diaphysis of the femur. The bottom row shows isotype controls for respective stains. Expression along the endosteal surface is marked by closed arrowheads. Expression of protein aro [file 13287_2024_3786_MOESM1_ESM.pptx]

## Slide 1
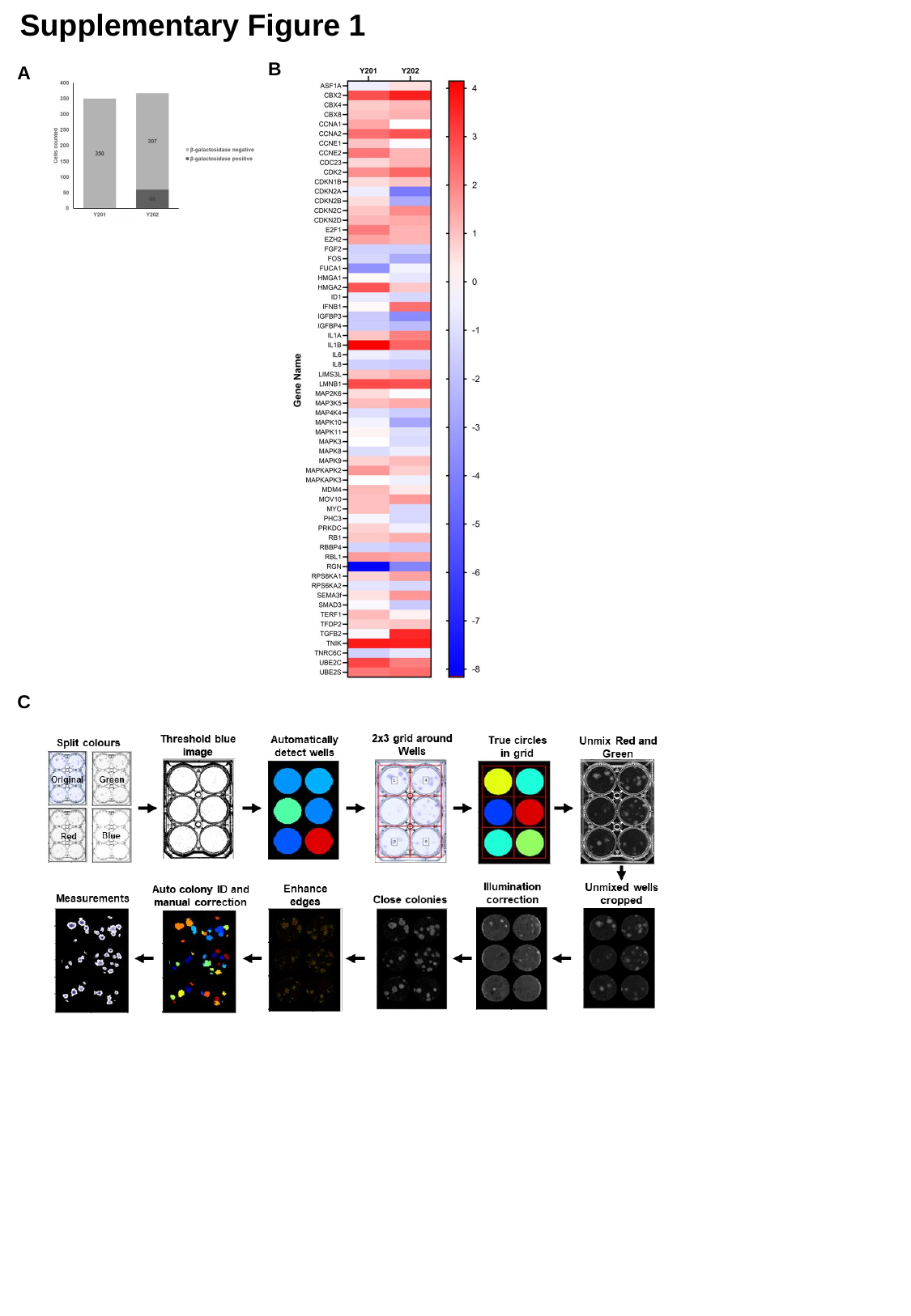

Supplementary Figure 1
B
A
C

## Slide 2
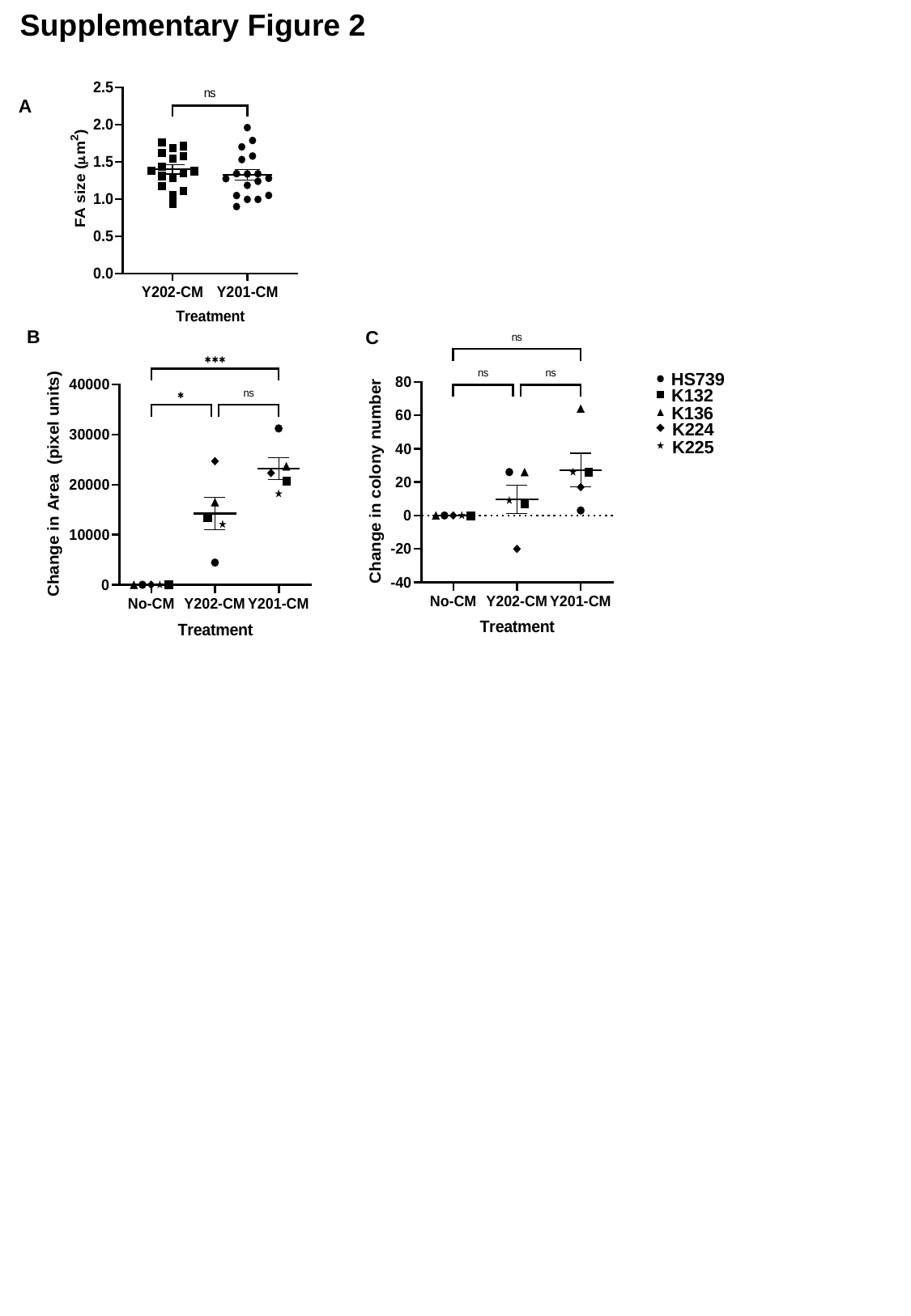

Supplementary Figure 2
A
HS739
K132
K136
K224
K225
B
C

## Slide 3
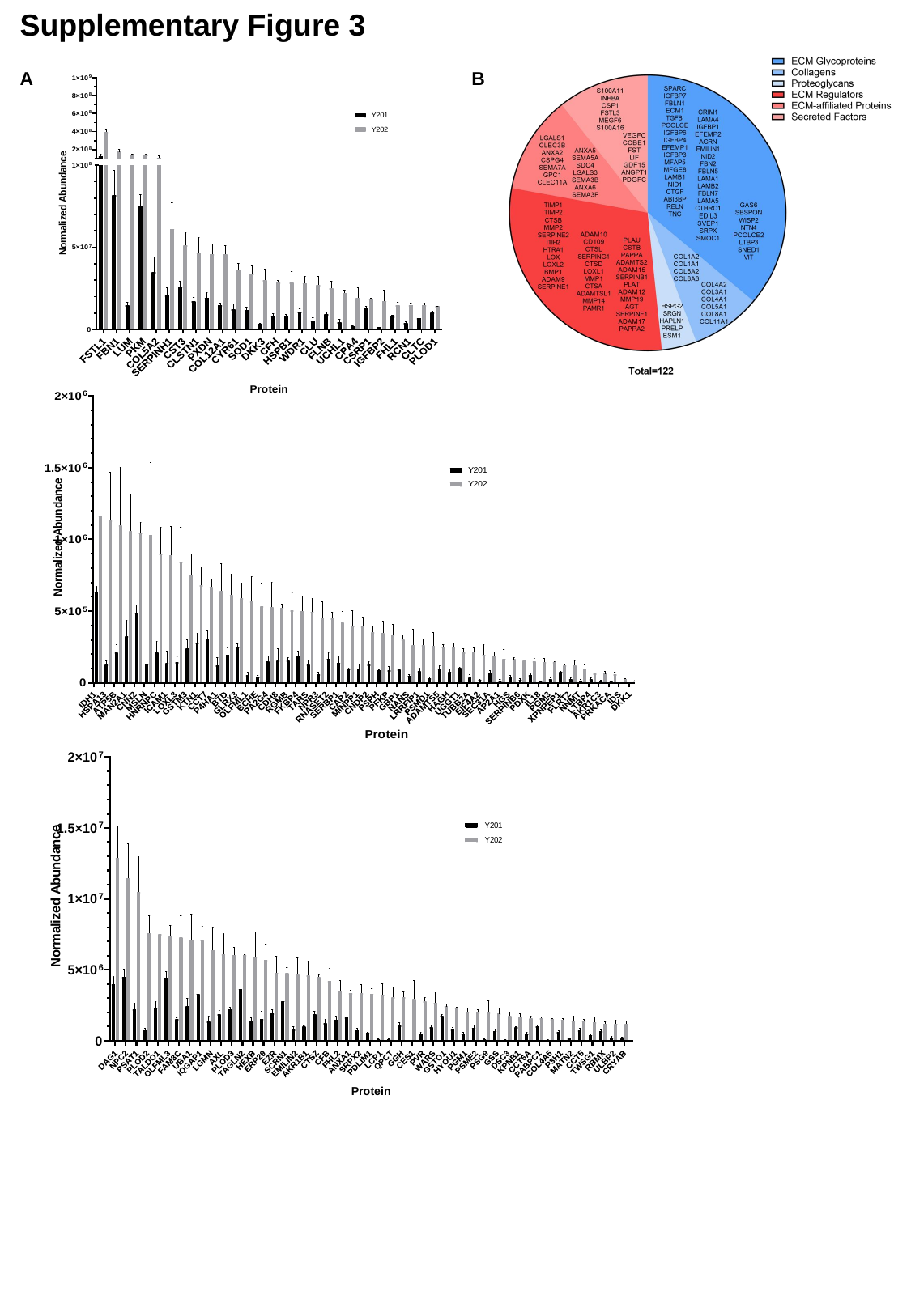

Supplementary Figure 3
A
B

## Slide 4
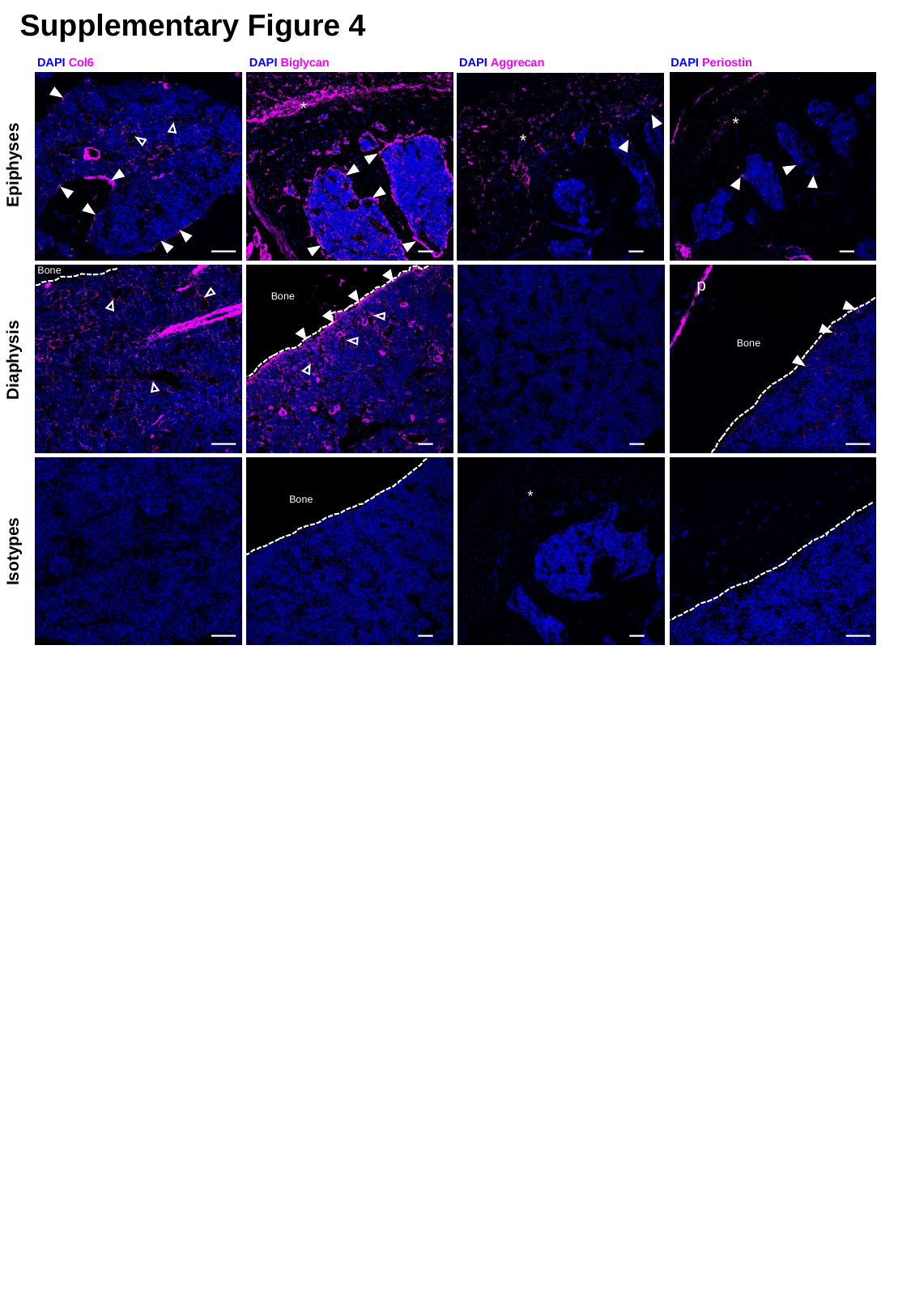

Supplementary Figure 4
DAPI Col6
DAPI Biglycan
DAPI Aggrecan
DAPI Periostin
*
*
*
Epiphyses
Diaphysis
*
Isotypes
Bone
p
Bone
Bone
Bone

## Slide 5
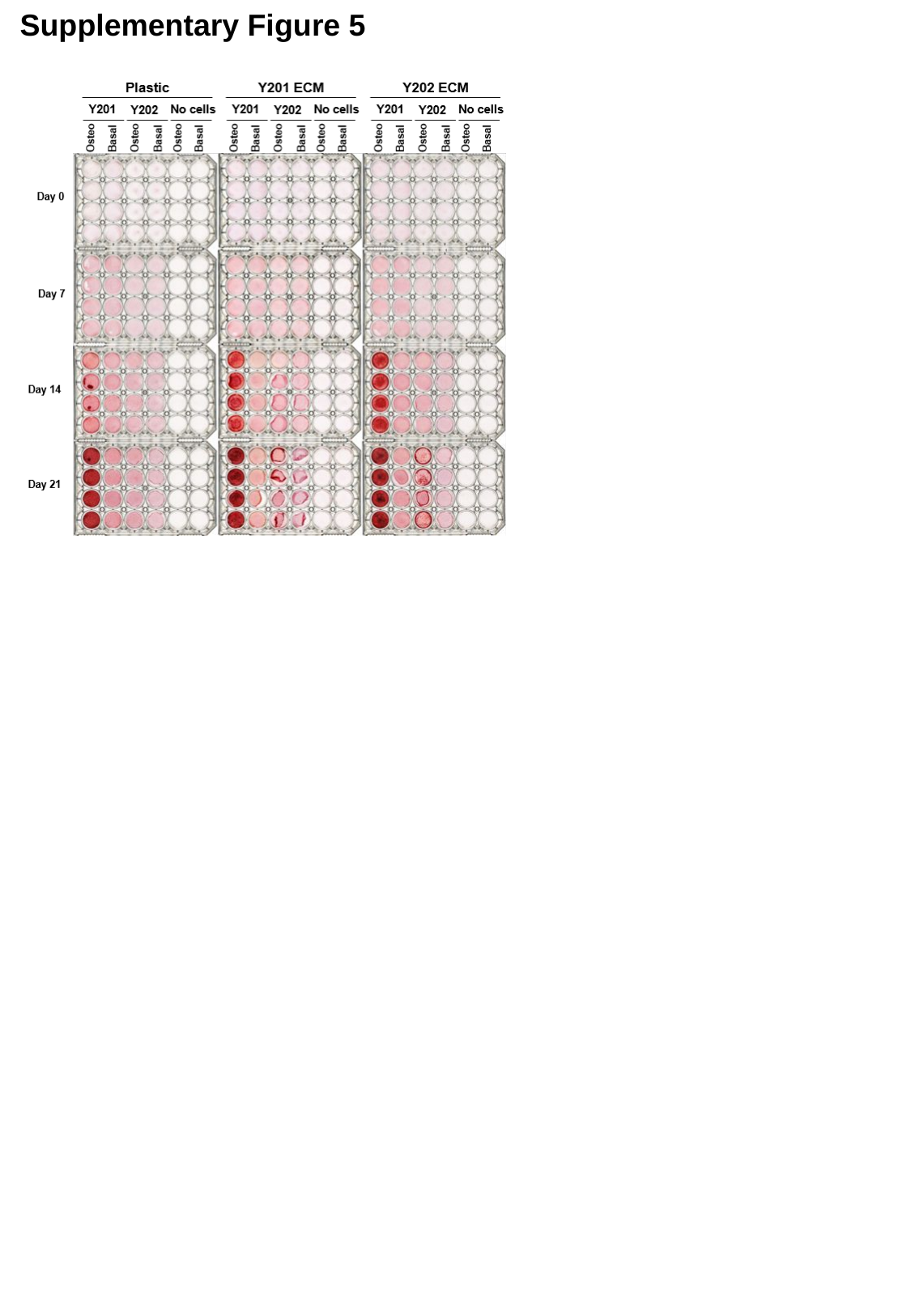

Supplementary Figure 5
