# Supplementary material for: Extracellular matrices of stromal cell subtypes regulate phenotype and contribute to the stromal microenvironment in vivo
Source: Stem Cell Res Ther. 2024 Jun 18;15:178. doi: 10.1186/s13287-024-03786-1 (PMC11184721; doi:10.1186/s13287-024-03786-1)
Supplement: Supplementary file 2 — Supplementary Table 1: Antibodies used for all immunofluorescence assays. [file 13287_2024_3786_MOESM2_ESM.docx]

| **Type** | **Target** | **Clone/Cat. No** | **Manufacturer** | **Host Species and isotype** | **Conjugate** | **Dilution** |
| --- | --- | --- | --- | --- | --- | --- |
| Primary | Aggrecan | 13880-1-AP | Proteintech | Rabbit | - | 1 in 50 |
| Primary | Biglycan | 16409-1-AP | Proteintech | Rabbit | - | 1 in 200 |
| Primary | Collagen VI | AB6588 | Abcam | Rabbit | - | 1 in 200 |
| Primary | Periostin | AB14041 | Abcam | Rabbit | - | 1 in 100 |
| Primary | Vinculin | V9131 | Sigma-Aldrich | Mouse IgG1, κ | - | 1 in 400 |
| Primary | CD271 | ME20.4 | BioLegend | Mouse IgG1, κ | - | 1 in 100 |
| Secondary | Rat IgG | A11077 | ThermoFisher | Goat | AlexaFluor 568 | 1 in 300 |
| Secondary | Rabbit IgG | A21244 | ThermoFisher | Goat | AlexaFluor 647 | 1 in 300 |
| Secondary | Rabbit IgG | A11011 | ThermoFisher | Goat | AlexaFluor 568 | 1 in 300 |
| Secondary | Mouse IgG | A11001 | ThermoFisher | Goat | AlexaFluor 488 | 1 in 300 |
| Isotype control | - | Poly29108 | BioLegend | Rabbit | - | Matched to primary |
| Isotype control | - | eBR2a | eBioscience | Rat IgG2α, κ |  | Matched to primary |
| Isotype control | - | MPC-11 | Biolegend | Mouse IgG1, κ |  | Matched to primary |

**Supplementary table 1. Antibodies used for all immunofluorescence assays**
